# Anger as a Basic Emotion and Its Role in Personality Building and Pathological Growth: The Neuroscientific, Developmental and Clinical Perspectives

**DOI:** 10.3389/fpsyg.2017.01950

**Published:** 2017-11-07

**Authors:** Riccardo Williams

**Affiliations:** Dipartimento di Psicologia Dinamica e Clinica, Sapienza Università di Roma, Rome, Italy

**Keywords:** basic emotions, anger, motivation, psychodynamic, development, affective neuroscience, personality disorders

## Abstract

Anger is probably one of the mostly debated basic emotions, owing to difficulties in detecting its appearance during development, its functional and affective meaning (is it a positive or a negative emotion?), especially in human beings. Behaviors accompanied by anger and rage serve many different purposes and the nuances of aggressive behaviors are often defined by the symbolic and cultural framework and social contexts. Nonetheless, recent advances in neuroscientific and developmental research, as well as clinical psychodynamic investigation, afford a new view on the role of anger in informing and guiding many aspects of human conducts. Developmental studies have confirmed the psychophysiological, cognitive and social acquisition that hesitate in the pre-determined sequence appearance of anger and rage in the first 2 years of life. The so-called affective neurosciences have shown the phylogenetic origin of the two circuits underlying the emergence of anger along with its evolutionary role for promoting survival. This view has been integrated by the psychodynamic theory of motivational systems that attribute a double role to anger: on the one hand, this affect works as an inwardly directed signal concerning a pressure to overcome an obstacle or an aversive situation; on the other hand, anger is also an outwardly directed communicative signal establishing differentiation and conflict within interpersonal relationships and affective bonds. Of course, human peculiar mental functioning requires the appraisal of such signals by higher cortical functions and, there is little doubt that the meaning that orientates individual behaviors is, eventually, construed on a social and cultural level. At the same time, everyday life experiences as well as clinical insights into psychopathic, narcissistic and borderline personality pathology clearly illustrate the necessity to correctly interpret and give answers to the basic questions raised around the topic of anger as a basic emotion.

## Introduction

As widely discussed by the Editors of this volume, the basic emotions theory (BET) has undergone a series of important criticisms that question their prominent role in human affective experience. In this paper, it will be argued that the new framework of motivational systems allows to acknowledge some aspects of the criticisms to BET, while bolstering its role in the understanding of personality building and psychological functioning. The general arguments in support of BET as a core aspect of motivational processes will be further illustrated through the presentation of some clinical phenomena in which the alterations of the mental processing of anger as a basic emotional signal play a pivotal role. As a beginning, the criticisms of the notion of BET could be summarized into the four following points.

(a) The description of everyday human mental life shows that the variety of affective experience can hardly be reduced to the activation of the single units of analysis described by BET. Emotional experiences seem more nuanced, fluid, cognitively sophisticated and not so discontinuously compartmentalized as BET seems to presuppose (Stern, 1985).
(b) BET falls short in explaining the role of experiences of learning and sociocultural influences on shaping the modes of expression, variety of meanings and possible functions of affective experiences. For instance, although the emotions of hate, jealousy or envy can be labeled as negative experiences potentially leading to aggressive intentions toward co-specifics and possibly including the basic emotion of anger, they can be hardly considered as primary, universally spread emotions (Solomon, 1984; Harrè, 1986). The contents of such emotions are more easily understood as a product of cultural conceptions concerning the notions of identity, guilt, property, sexual and sentimental interactions. Research as well as anecdotic evidence highlighted the diverse intensity and diffusion of such emotions between different social contexts, thus confirming the influence played by culture in generating such mental experiences (Rosado, 1984).
(c) While BET is founded upon the phylogenetic roots of basic emotions (namely, cross-species analogies of the emotional manifestations), some authors have recently questioned the fact that the cross-species schemes of activation commonly referred to as basic emotions can be labeled as emotions at all. For instance, LeDoux (2016) argues that these primary systems of response do not enter the domain of emotional experience until they are secondarily represented by higher cognitive systems. In this sense, the specific content of emotional experience cannot be directly regarded as the simple product of the activation of the basic schemes of response. Furthermore, the real survival meaning of basic emotions is highly reduced in an environment in which external threats are decreased and adaptation is more and more dependent upon group interactions and highly sophisticated cognitive operations. Human emotional experience is pervasive and not limited to moments of external changes, but most often it originates from inner contents such as fantasies, imagination, memories.
(d) Contrary to what required by BET, developmental as well as psychophysiological research data do not support the view of the existence of neatly distinguishable categorical expressions and manifestations of emotions. Some emotions such as fear are undoubtedly evident from the first year of life, but this is not the case for other emotional categories, such as, for instance, shame, or anger (Sroufe, 1995; Ekman, 1999). Individual differences in the expression of emotions show how some people hardly exhibit the entire gamut of categorical emotions considered by BET. For instance, children exhibiting very cautious and coy attitude do not engage in episodes of rage at their peers or parents (Natsuaki et al., 2013). Moreover, some of the basic emotions more easily detectable in the early phases of development cannot be observed in later stages of life. Research data often failed to evidence the existence of specific patterns of psychophysiological modifications supposedly underlying BET (Scarpa and Raine, 1997; Scarpa et al., 2010).

In this paper, it will be argued that, although correct, some of the criticisms aimed at BET can be overcome by reframing the evolutionary meaning of BET within the broader notion of motivational systems. In particular, the convergence of developmental, psychodynamic and neuroscientific view of emotion and motivation affords a new perspective in which not only the notion of basic emotions results scientifically viable, but it also shows its central function for the understanding of human emotional life.

## The Communicative And Behavioral Approaches To Basic Emotions

Since Darwin’s pioneering investigations (Darwin, 1872), universal emotional heritage of the human species has been conceived in terms of its value for survival. In their original interpretation, etholigists understood the maintenance of some basic schemes of automatic response named emotions as a way to increase survival by facilitating the communication between co-specifics (Ekman, 1992). The ritualization of instinctive behaviors into fixed patterns of facial expressions, postures, and gestures was thus believed to serve as a message signaling to other members of the group one’s own behavioral intentions or reactions evoked by an unknown environmental condition (Ekman, 1999). For instance, the display of teeth originally precedes an attack, but its ritualized version contained in smile, in fact, indicates the freezing of an aggressive intent and, therefore, the manifestation of a friendly overture (Lorenz, 1978). In a following theoretical interpretation of the survival functions of basic emotions, the stress was placed on their preparatory role within instinctive schemes of response highly necessary for quick adjustment to an unpredictable and quickly modifying environment. The activation of the behavioral and psychophysiological modifications observed during emotional experiences was considered as a part of an automatic response (selectively elicited by specific environmental cues) prompting and preparing the whole organism to the most suitable adaptive behavior. The need to promptly react to threats to survival or sudden environmental changes was, of course, not limited to inferior species, but it was considered a fundamental adaptive prerequisite also for superior mammalians, of which brains would be capable of much subtler analyses of the stimuli. This justifies the persistence of such rough level of response in our species and its complex interactions with the most recently evolved and more sophisticated information processing modes of our brain. Such view of emotions as parts of wider schemes of automatic, unconscious, and fast adaptive systems of response is now spread to the whole psychological field as well as to current neuroscientific literature (Gazzaniga, 2008). Despite this progressive modification of BET, many authors would consider the key critical points presented in the introduction still true for these new evolutionary views of human emotional life.

## Reframing The Basic Emotions Theory In Motivational Systems Approach

A general reconsideration of the meaning of basic emotions has been recently proposed within a motivational perspective drawing on contributions from the study of animal instinctive behavior and the psychodynamic perspective. The contributions coming from modern ethology was used to review the psychodynamic view of human development and placed basic emotions at the core of motivated behavior. Modern ethology relied on cybernetics to reinterpret instincts in terms of goal corrected behavioral plans that flexibly (unlike the fixed behavioral sequence previously meant to characterize instincts) employ inborn or acquired motor patterns in order to achieve an expected outcome enhancing individual fitness (Hinde, 1974). In its original formulation the notion of behavioral system helped reshaping the theory of human motivation, coherently with the Darwinian perspective on the inborn tendencies ruling intentional behaviors (Rosenblatt and Thickstun, 1977). Bowlby fully drew on this new ethological framework to propose the existence of an innate goal for human infants (as well as other primates) to establish and maintain optimal proximity to the caregiver, which is, the attachment behavioral system (Bowlby, 1969). However, Bowlby’s perspective only secondarily considered the role of affective experience (separation anxiety) in regulating goal corrected behaviors.

### From the Behavioral Systems to the Motivational Systems: The Contribution of Psychodynamic Theory to Basic Emotions

A more recent proposal deriving from ethological and developmental literature was introduced within the psychodynamic perspective by Lichtenberg (1989). Lichtenberg proposed to modify the construct of behavioral systems into the notion of motivational systems. Any motivational system, just like any behavioral system, is goal directed (evidently any goal of each motivational system is fixed by evolution yielding some important gain for individual and species survival). More specifically, however, motivational systems are not meant to mechanically work as a plan of behavior unfolding through a constant perceptive feedback that matches the actual behavior with the set goal. Any motivational system is regulated by a single affect, the associated representations, memories and plans of behavior. Lichtenberg (1989) argued that each motivational system originally possessed a specific affective signal that is able to orientate the human behaviors toward the set goal easily observable from the first months of life. It should be observed that in its original proposal Lichtenberg did non-explicitly referred to the basic emotions traditionally studied by the BET. However, he included some of the classic basic emotions, such as fear and anger, among the ones regulating his five motivational systems. The specific affect is responsible for (a) the activation of the motivational system, (b) the retrieval of the relevant representations guiding the behavioral plan, (c) signaling the eventual achievement or failure of the expected outcome. Interestingly, the avoidance or maintenance of each specific affective state becomes the inherent goal of the motivational system.

In order to achieve the affective goal of the motivational system, the set of stored representations concerning past experiences related to any specific affective state is activated, and current behavior is planned consistently with those representations. In the course of development, interpersonal experiences, cognitive development and cultural meanings can intervene to modify the early interactive representations pertaining each motivational systems, but their affective core remains unchanged (Lichtenberg et al., 2011) The actualization of representations of past events in the present context of experience constitutes the motive defining the content and aims of current behaviors and perceptions (Lichtenberg, 1989). Therefore, specific affective states, by creating single motives, represent the linkage between the goals fixed by the species’ evolutionary history and the individual’s actual mental experience. In this perspective, basic emotions are what, in fact, connect evolutionary set goals to individual motives leading behaviors and creating personal meanings in everyday lives. In this complex architecture of motivated behavior, basic emotions can easily account for the variety and plasticity of performances through which humans achieve their basic evolutionary goals. Furthermore, the leading role of affects renders motivational processes open to learning, cognitive refinement and cultural contributions to individual biological adaptation.

### Affective Neurosciences and the Survival Systems

Notwithstanding some important theoretical differences, neuroscientific approaches are basically resonant with psychodynamic notion of motivational systems and provide further implications for the role of basic emotions in human behaviors. In the first place, current neuroscientific approaches evidenced that decision-making and motivated behaviors are supported by activation of neuroanatomical structures that are devoted to the detection of specific signals relevant for individual survival (MacLean, 1990; Panksepp, 1998). Such sub-cortical structures are responsible for fast responses that have maintained an important adaptive role, despite the emergence in the evolutionary history of more refined ways of stimulus analysis and behavioral adaptation. In particular, each neuroanatomical structure is held responsible for responses to conditions that involve organismic homeostatic needs and reproductive functions. Furthermore, in the course of evolution these systems of fast adjustment gradually included social behaviors that have a direct impact on survival through group interactions (e.g., attachment, friend/foe reactions, empathy).

A second important contribution from neuroscientific research evidenced that the neuroanatomical sites of basic emotions virtually coincide with the ones of the more ancient schemes of behavioral adaptation (Panksepp, 1998). This evidence led many researchers to incorporate basic emotions into the so-called survival systems, basic behavioral response systems that guarantee the preservation of individual integrity in the face of sudden changes in the internal and external milieu of adaptation (LeDoux, 2016). Basic emotions such as anxiety, anger, fear can thus be regarded as fragments of a wider pattern of behavior leading to an immediate adaptive response to environmental conditions that represent a threat/opportunity for individual survival. This view substantially equals the classic theory of basic emotions as systems prompting fast adaptive behavioral reactions. However, a more complex analysis of the survival systems of which basic emotions are now part, allows researchers to provide a more nuanced picture of the motivational processes underlying human behaviors.

Indeed, a third important contribution coming from recent neuroscience of motivation sees basic emotions not only as a part of the innate quick responses to threat to survival. The evolution of more recent cortical brain structures created the opportunity to overcome and, possibly, improve the strategies of behavioral adaptation of the more ancient survival systems. This improvement was pursued by evolution via the amelioration of the specificity of perceptive analysis of stimuli (including symbolic and linguistic categorization), the comparison of present conditions with previous experiences (new memory systems), the higher specialization and refinement of behavioral responses and, what is particularly true for human beings, the role of learning and cultural transmission (LeDoux, 2016). Differently from what it may appears, however, the role of more basic systems of response is neither discarded nor diminished by this achieved complexity (Panksepp and Biven, 2012). Although the presence of basic affective responses may be only a part of our subjective experience, the basic emotions linked to the survival systems are the raw material which the more sophisticated analyses carried out by superior centers of the brain are founded upon. This motivational view goes actually beyond the traditional communicative and behavioral interpretation of basic emotions, stressing the evaluation and informational role basic emotions play in complex decision making processes. Of course, the interpretation of such signals is not carried out automatically and does not lead to a simple and self-evident translation of what is going on within our bodies.

As both psychodynamic theory and neuroscientific approaches evidenced, the basic emotional responses are dynamically interwoven, fluidly change and, to some extent, may be employed by different motivational systems. Furthermore, the interpretation of the bodily cues that we rely on to interpret the outside world much depends on the nature and modes of storing of previous experiences and, through development, is gradually influenced by interpersonal and cultural processes. It is this complex “working through” of internal bodily experiences that creates the multicolored and multifaceted nature of our emotional experience. In this sense, the old version of BET is correctly criticized for its reductionist approach to human affective experience seems totally shareable. However, it should be remembered that there would not be a conscious experience of oneself without the interpretation of the basic affective traces. The building of personal meaning as well as the experience of being a subject (a “person”) could not be accomplished in the absence of the deciphering and interpretation of the bodily signals pertaining the survival systems (Modell, 2003; Northoff et al., 2011). More importantly, the way evolution has allowed us to establish and maintain a strong connection between our mental functioning and our basic organismic and social needs is through the processing and elaboration of basic emotions. Surely, it is not possible to state anymore that we are “driven” by basic emotions and instinctual forces in the old psychoanalytic or ethological sense. Similarly, it may be argued that even if the motor and autonomic components of basic emotions often represent the recognizable final pathway of expression our affective experience, our adaptation does not depend as much on these basic emotional responses as on more complex and more rational behavioral strategies, social interactions and cultural cooperation. Anyway, it should be considered that our perception of the world as well as our behaviors would be meaningless without a constant and adequate work of interpretation of our basic emotional experience. Psychodynamic thinking has recently paid much attention to the bridge to be built between the instant raw metaphors created by the fragmented emobodied representations of the interpersonal world, and the imaginative interpretation, mediated by the symbolic processes, that confer a new and transformative meaning to such experiences (Modell, 2003). This new perspective allowed to reframe the problem of abnormal personality development in terms of failures and collapses of the representational systems designated to elaborate emotional and motivational signals (Fonagy et al., 2002). The analysis of some clinical phenomena related to the activation of the experience of anger and failures in its interpretation will hopefully shed some more light as to the relevance of this new model of BET.

## The Basic Emotion Of Anger

Anger has always been included in the repertoire of basic emotions, mainly given its distinct and universally recognizable pattern of facial expression (Ekman, 1999). Research has nonetheless evidenced some critical points that question the universal biological meaning of the emotion of anger and, therefore, the general relevance of BET in explaining the affective states possibly connected to this emotional state. First of all, the data indicating a specific psychophysiological profile of activation for anger seem still controversial. Psychophysiological parameters of anger are common to other emotional conditions, such as, for instance, a general condition of stress or fear or predatory behaviors (Scarpa et al., 2010). Researchers have found it difficult to find a specific place within the general categorization of positive and negative emotions (Watson et al., 2016). Anger entails a negative activation that leads the individual to resolve the tension through active behaviors. At the same time, behaviors sustained by anger can result in approaching behaviors usually sustained by positive emotions (Scarpa and Raine, 1997). Unlike other basic emotions, the environmental conditions expected to elicit anger are not invariably distinguishable as BET would require (Ekman, 1992). Anger can appear as a reaction to a condition of bodily distress, as a way to protect oneself to an attack from a predator (in this sense, anger is a possible consequence of fear; Wilkowsky and Robinson, 2010), as an emotion supporting goal-directed behavior when a circumstance in the outside world prevents the desired goal to be fulfilled, causing frustration (Panksepp, 1998). Another cardinal aspect of BET, which is, the social impact of the display emotion on other people is also controversial in the case of anger. Facial expression of anger can be interpreted as a sign of aggression, inducing reactions of fears or proneness to engage in a conflict, or can otherwise elicit enlivening feelings of sharing in other subjects, depending on the evaluation of the context (Emde, 1984). Critics of BET also highlighted that the expression of anger is virtually totally inhibited in some cultural contexts (Rosado, 1984). In a similar vein, it is stressed that some affective or motivational states conceivably connected to anger, such as envy, jealousy, hate or the aggressive pursuit of a specific goal are not accompanied by the display or subjective experience of anger nor rage, as if these negative feelings and sentiments were culturally built (Harrè, 1986).

Overall, these controversial points do not rule out the possibility to consider anger as a basic emotion and to assign it a central role in our affective life. Again, a motivational analysis based on phylogenetic and ontogenetic considerations can improve our understanding of the relevance of anger as a basic emotional signal in our affective life. The analysis of the neuroanatomical structures implied in the expression of anger place its phylogenetic origin in a basic reaction to a condition of distress. Probably such reactions evolved as a response to a condition of physical constriction as an ultimate way for the individual to free itself from a predator or to an external condition causing pain or irritation. This basic scheme of response is located at a very deep level in the brain [the Peri Acqueductal Gray (PAG)] where other centers coordinating homeostatic responses are also situated (Panksepp and Biven, 2012). According to neuroscientific accounts, such basic protective role of the reaction of anger gradually evolved into a more complex sequence of response activated by the perception of a threat in the outer world and useful to initiate and support the fight-flight response. The integration of such complex response was guaranteed in the course of evolution by the interaction of centers that are placed in the amygdala (Panksepp and Biven, 2012). A further step in the evolution of anger is characterized by the recruitment of such basic reaction by the motivational system of goal attainment. The general circuit regulating the approaching behaviors toward a goal is regulated by the reward system. The motivation to achieve an objective is flexible and is able to adjust the behavioral plans according to the possible external obstacles as well as the internal sources of error. The psychophysiological activation typical of the reaction of anger can be called into play to help the organism overcoming the obstacles more vigorously and enduring the attempts at reaching the desired goal. The ancient reaction of anger is, therefore, recruited by several motivational systems through neuroanatomical connection that evolved later in evolution and define different possible fixed patterns of response resulting in the final emergence of anger. This instance of evolutionary expectation can clearly account for the variety of sources possibly eliciting anger reactions and highlights how such emotional response is a fundamental part of the adaptive repertoire shown by human beings.

### The Ontogenesis of Anger

The ontogenesis of anger reactions can further explain how such basic emotion becomes a necessary aspect of the sophisticated emotional life of the individual. Developmental researchers showed that a proper expression of anger does not appear until the last months of the first year of life (Sroufe, 1995). Before then, only a less specific reaction of distress and irritability can be observed, hardly distinguishable from other negative reaction such as crying, hunger, pain. The reaction of distress appearing in the first months of life is regarded as a basic response emerging in the presence of a sharp accumulation of psychophysiological activation, whether the source of this sudden increase in arousal is due to endogenous fluctuations of the nervous system or to the outside stimulation. The precursor of the reaction of anger is thus elicited by the specific psychophysiological parameters. It is only when, at the end of the first year, the infant becomes able to differentiate between means and ends of her behavior and to perceive distinctly that her intentional action is blocked, that a proper reaction of anger emerges. Notably, the emotion of anger makes its appearance when the child is able to attribute a psychological meaning to the stimulation (“there’s an obstacle hindering the achievement of a goal”). The psychological meaning attributed to the situation induces the same psychophysiological parameters able to elicit the previous reaction of distress (sharp increase in internal arousal). Subsequently, cognitive development and learning enable the child to anticipate the sources of frustration and to link her capacity to explore the environment to the capacity (possibly sustained by anger) to overcome the obstacles (Lichtenberg, 1991). As self-awareness and social awareness come to dominate the child’s psychological judgments, anger is finally directed at other people or at herself, finally acquiring the form of what is commonly acknowledged as rage (Sroufe, 1995). Through further cognitive and social growth, the psychological meaning of rage is, of course, more and more molded by interpersonal experience and shared cultural notions becoming a central aspect of interpersonal conflict negotiations. Anger and rage are then transformed into feelings of hate, competition, subtle resentment, sadism, contempt, envy, jealousy, possessiveness. This variety of sentiments are initiated by the appearance of rage, but they become more and more differentiated and partially detached from this basic emotion (Parens, 2008). Of course, as we shall see in the next paragraph, the degree to which rage become a part of the individual way to exert one’s control upon the external world, conflict management and social assertiveness is much influenced by the actual social experience and interpretations that the caregivers offer to the child’s behaviors as well as by the wider social context of social norms and established meaning. It is noteworthy, that these ontogenetic advancements, no matter how complex the expression of anger may result, could not take place without the recruiting of the basic schemes of response gradually evolving in the first 2 years of life.

## Personality Building And The Metabolism Of Anger/Rage

Recent developmental as well as clinical accounts highlighted the importance of anger and rage for normal and abnormal aspects of personality growth. The expression of anger is regarded as a prerequisite in the acquisition of exploration of the environment (Mahler et al., 1975; Sroufe, 1995), achievement of goals and behavioral plans (Stechler and Halton, 1987), establishment of the sense of personal control over one’s own actions, conflict negotiation (Lichtenberg, 1989), defense of personal integrity (Modell, 1993), differentiation of personal vs. other’s personal motives and points of view (Parens, 2008). As discussed in this paper, it is evidenced that in the first years of life the basic emotion of anger, regardless of its phylogenetic origin and its previous ontogenetic precursors, is recruited in the service of the wider motivation to achieve a desired goal. Therefore, the possibility to resort to anger or rage is seen as a basic step (though not the only one, of course) to assert one’s own autonomy and a sense of mastery of the self and counterbalance feelings of shame and vulnerability, what psychoanalysts define as “healthy narcissism” (Ronningstam, 2005). Anger and rage are thus considered as necessary instruments to reestablish a feeling of personal consistency and autonomy or to endure in a goal pursuit when a failure is experienced (Mahler et al., 1975; Kohut, 1977). Naturally, assertiveness and the sense of autonomy and mastery of the self should not wholly be considered coincident with the expressions of anger and rage. Many other affective, cognitive and social acquisitions are the necessary underpinnings of the evolving sense of autonomy and narcissistic integrity. Furthermore, the healthy manifestations of anger should be tamed by feelings of empathy for the others, acknowledgment of their point of view and full appreciation of the nature of the affective relationship with them, as well as the respect of the ethical and social norms unavoidably constraining individual assertiveness and achievements. As a consequence, normal expressions of anger and rage should be distinguished from arrogance, special sense of entitlement, sadistic control, interpersonal exploitation, affective manipulation, and violence. Clinical literature evidenced that when proper limitations of the manifestations of anger systematically fail to occur we are in the face of personal abnormal development and risk of antisocial conducts. We shall now see how these distortions in personality growth are mainly due to two conditions implying the metabolism of the affective signal of anger: (a) the wrong and recurring processing of other motivational cues implying the normal recourse to anger or rage (e.g., fear, frustration, wound to personal integrity) leads the individual to repeated or exaggerated expressions of this basic emotional manifestations; (b) the individual has learnt to confuse the internal signal of anger and the behavioral manifestations of rage with her own self-assertion, affirmation of autonomy, sense of personal control and integrity.

### Hyperactivation of the Anger/Rage Signals in Borderline Personality Disorders and Psychopathy

Diagnostic approaches (Kernberg, 1984; Gunderson, 2001; American Psychiatric Association [APA], 2013) always identified unrestrained and frequent bouts of rage as one of the key clinical features of Borderline Personality Disorder (BPD). Most often these intense feelings of rage can also give rise to dramatic self-harming behaviors, possibly hesitating in suicide attempts. The dysphoric background characterized by irritability and anger is also held responsible for the affective instability and fragmented sense of identity characterizing BPD patients (Gunderson, 2010). In order to maintain at least a form of positive relatedness with the meaningful others and a sense of personal worthiness and autonomy, the BPD patients are supposed to split the angry and raging aspects of their personality from their self-representation and from the experience of their relationship with outer world (Kernberg, 1975). BPD patients are reported to experience such an unbearable amount of anger given their proneness to perceive personal threats in the outside world, mainly in close relationships, owing to both temperamental factors (New et al., 2008; Gunderson, 2010) and early traumatic experience in the attachment matrix (Chiesa et al., 2016). As a result, anger reactions are easily elicited as a basic defensive (flight-fight) response to the feeling of being attacked. At the same, time the fragile sense of self and extreme dependency from the meaningful other in which the BPD patients feel entrapped, often lead these patients to transform outward manifestations of rage into self-harming or passive aggressive conducts (Kernberg, 1975). Another example of mistaken processing of environmental or motivational clues resulting in overly recourse to anger is represented by the clinical phenomenon of psychopathy. Anger and rage do not characterize the core of this rare and extreme condition, although expressions of anger and rage may be frequently associated to it Blair (2009) and Glenn and Raine (2014). The emergence of anger and rage in psychopathic patients has been recently explained through a peculiar failure in the processing of negative reinforcement (Blair, 2009). Psychopathic patients show a deficit of functioning in the area of the brain designated to the detection of failure of behavioral plans. When the execution of any behavioral plan does not obtain the expected result the anterior cingulate cortex signals the pre-frontal cortex to adjust the behavioral plan in order to achieve the goal. If the cingulate cortex is not activated by the negative outcome, as happens in the case of psychopathic patients, the original behavioral plan is carried out over and over again. Quite opposite to BPD patients, individuals with psychopathic traits are reported to underestimate the impact of negative emotions (Masi et al., 2014). Since reactions of rage and anger are unavoidably engendered any time the execution of the behavioral plan fail to achieve its goal, psychopathic individual are over-exposed to thy stipe of angry reactions. In this latter case, therefore, the basic emotions of anger are sustained by the reward system and are expressed in their purest form because this system is not adequately supported by the emotional information processing of the negative outcomes of the subject’s behavior. This inadequate processing is responsible for both the perseverance of behavioral efforts, neglecting the interpersonal consequences, and the associated angry reactions.

### The Role of Anger/Rage Signals in Narcissistic Personality

The case of Narcissistic Personality Disorder affords a different view of an inappropriate recourse to anger and rage affective expressions. According to Kernberg (1975) the core of narcissistic personality pathology is represented by the fusion between the attempts at establishing a primitive grandiose sense of self and the expression of anger. In a few words, the narcissistic individual mistake her self-assertiveness and sense of personal worth with the aggressive control of the meaningful other’s appreciation and mirroring. The arrogance and exaggerated sense entitlement, the intense reaction of rage to any perceived threat to their self-esteem or to the frequent feelings of shame, exploitative or even sadistic behaviors portraying the most recognizable forms of narcissistic personality are the result of this basic confusion: to assert oneself and to protect a vulnerable self-esteem, it is necessary to be feeling enraged and in aggressive control of the relationship. When such attempts at control fail, the narcissistic patient tries to protect herself from the ensuing intense feeling of shame through rage (Kohut, 1977; Ronningstam, 2005). Clinical literature showed how the narcissistic patients’ personal history is often sprinkled with interpersonal relationships that denied a full and deep recognition of the patients’ early sense of autonomy and personal differentiated existence. Most frequently, as a child, the narcissistic patient was humiliatingly treated as an extension of her own parents or was recognized only for her superficial appearance or talents (Fernando, 1998), developing only a scarce capacity for emotional self-recognition and self-regulation. In some other cases, a particular temperamental endowment, namely, an overly need for reward and affective gratifications exposed these patients to extreme feelings of shame and personal failure. The diverse manifestations observed in the widely acknowledged distinction between the Vulnerable and Grandiose forms of narcissistic pathology (Pincus and Lukowitsky, 2010) seem to shed a clear light on the importance of the processing of anger and rage in this area of personality pathology. In the Grandiose variant, the narcissistic patient has learnt that anger and aggressive control over his interpersonal environment is the equivalent of personal empowerment, autonomy and internal consistency. A general strategy of regulation of self-esteem and self-enhancement stems from this equation, that leads to manifestations of entitlement, arrogance, manipulativeness and interpersonal explotativeness, direct sadistic or aggressive attempts to exert a control on others’ states of mind and behaviors. The key importance of the basic emotion of anger in narcissistic personality is also testified to by the vulnerable variant of this pathology of character that, explicitly, is dominated by a pervasive sense of shame, inadequacy and personal failure (Pincus and Lukowitsky, 2010). These otherwise called *covert* or shy (Ronningstam, 2005) forms of narcissistic pathology are reported to be very wary of any manifestations of anger and rage. When these affective reactions take place, the covert narcissist may fail to acknowledge it or the reasons why they occurred to them. These patients, in fact, prefer to stave off any feeling connected to personal assertiveness in order to hide their grandiose expectations and avoid possible subsequent frustrations and feelings of shame (Pincus and Lukowitsky, 2010). However, empirical and clinical evidence showed that the vulnerable narcissistic patients may be even more prone to resort to aggressive acts or antisocial conducts (Fossati et al., 2014). Indeed, when the more vulnerable patients are overpowered by emotions that prevent them from understanding their frustrated needs for assertiveness and the ensuing aggressive feelings, they become less able to modulate their behavior and understand its impact on other’s well-being (Baskin-Sommers et al., 2014; Lee-Rowland et al., 2017). In these two forms of narcissistic pathology, thus, the hyper-estimate and hypo-estimate of feelings of anger are two basic processes around which the peculiar strategies of this personality pathology revolve around. A final aspect to be taken into account with regard to narcissistic functioning and the processing of anger and rage, is represented by suicide. As a matter of facts, the clinical understanding of the narcissistic background for suicidal ideation and suicide highlighted the pivotal role of affective states imbued with feelings of hatred, sado-masochistic dynamics and revenge (Ronningstam et al., 2008). In this regard, one key step leading to suicide in narcissistic personalities is thought to be the denial of aggressive feelings engendered by narcissistic injuries. Strong defenses, often of a dissociative nature, against such aggressive and domineering attitudes toward meaningful others and the outer world in general, take place to hide and avoid threats to the personal sense of omnipotence and self-esteem. However, when such defenses fail this purpose owing to serious life events, the split-off angry and rageful feelings sustaining the sense of control and power are exacerbated and press the individual to action. The attack against the self escalating in suicidal conducts is the way such needs for aggressive control are expressed out of personal awareness, allowing for the restoration of the sense of mastery through the disguised fantasy of retaliation against the others who will remain alive.

## Conclusion

The many attempts to get rid of BET face a basic obstacle. In its purest form BET was a way to conceive of the influence of the evolutionary heritage and survival needs on human mind. No matter how convincing and effective the criticisms are to the single aspects of BET, its original message cannot be overestimated. In this paper, it was proposed that a new motivational framework for basic emotions allows to expand their role in affective experience and decision making processes. Neuroscientific, developmental and psychodynamic approaches all seem to point to an interpretation of basic emotions as systems of evaluation that work as internal signals orienting and giving meaning to our intentions and subjective experience. The introduction of a motivational point of view for basic emotions seems necessary, in order to consider how these basic systems of response can be transformed by more refined cognitive operations into diversified emotional contents. Furthermore, the motivational approach affords a new view in which basic emotions disclose its importance for human beings through interpersonal and cultural experiences.

## Author Contributions

RW is entirely responsible for the development of the ideas and theoretical research contained in this paper. The author is also entirely responsible for the realization and revision of the draft of the manuscript.

## Conflict of Interest Statement

The author declares that the research was conducted in the absence of any commercial or financial relationships that could be construed as a potential conflict of interest.
